# Targeted Disruption of the Intracellular Domain of Receptor FgfrL1 in Mice

**DOI:** 10.1371/journal.pone.0105210

**Published:** 2014-08-15

**Authors:** Gilles Bluteau, Lei Zhuang, Ruth Amann, Beat Trueb

**Affiliations:** 1 Department of Clinical Research, University of Bern, Bern, Switzerland; 2 Department of Rheumatology, University Hospital, Bern, Switzerland; University of Sydney, Australia

## Abstract

FgfrL1 is the fifth member of the fibroblast growth factor receptor (Fgfr) family. Studies with FgfrL1 deficient mice have demonstrated that the gene plays an important role during embryonic development. FgfrL1 knock-out mice die at birth as they have a malformed diaphragm and lack metanephric kidneys. Similar to the classical Fgfrs, the FgfrL1 protein contains an extracellular part composed of three Ig-like domains that interact with Fgf ligands and heparin. However, the intracellular part of FgfrL1 is not related to the classical receptors and does not possess any tyrosine kinase activity. Curiously enough, the amino acid sequence of this domain is barely conserved among different species, with the exception of three motifs, namely a dileucine peptide, a tandem tyrosine-based motif YXXΦ and a histidine-rich sequence. To investigate the function of the intracellular domain of FgfrL1, we have prepared genetically modified mice that lack the three conserved sequence motifs, but instead contain a GFP cassette (FgfrL1ΔC-GFP). To our surprise, homozygous FgfrL1ΔC-GFP knock-in mice are viable, fertile and phenotypically normal. They do not exhibit any alterations in the diaphragm or the kidney, except for a slight reduction in the number of glomeruli that does not appear to affect life expectancy. In addition, the pancreas of both FgfrL1ΔC-GFP knock-in and FgfrL1 knock-out mice do not show any disturbances in the production of insulin, in contrast to what has been suggested by recent studies. Thus, the conserved motifs of the intracellular FgfrL1 domain are dispensable for organogenesis and normal life. We conclude that the extracellular domain of the protein must conduct the vital functions of FgfrL1.

## Introduction

Fibroblast growth factor receptor-like 1 (FgfrL1) is a member of the fibroblast growth factor receptor (Fgfr) family [1]–[4]. It is expressed in nearly all tissues, but its biological function appears to be restricted to a very limited number of organs as demonstrated by studies with knock-out animals. FgfrL1 deficient mice develop quite normally to term and are born alive, but they die immediately after birth due to a malformed diaphragm muscle that is too weak to inflate the lungs [5], [6]. In addition, these mice lack both metanephric kidneys [7] and show slight abnormalities in the skeleton, primarily in the skull [6], [8]. The molecular mechanisms, by which FgfrL1 controls development of the diaphragm and the metanephric kidneys, are not known. It has been shown that the receptor is expressed in myoblasts, at particularly high levels in those that are about to differentiate into myotubes and myofibers [5], [9]. During development of the kidney, it is expressed in the metanephric mesenchyme at regions that are close to differentiating into epithelial renal vesicles [7], [10]. In knock-out animals, this mesenchymal-to-epithelial differentiation fails and no renal vesicles are formed.

All Fgfrs are type I transmembrane proteins, comprising an extracellular part with three immunoglobulin-like domains, a single transmembrane domain and an intracellular part [11]. The extracellular domain of FgfrL1 shares up to 50% sequence similarity with the corresponding regions of the other Fgfrs [4]. It interacts with heparin [12], [13] and with Fgf ligands, primarily with Fgfs 2, 3, 4, 8, 10 and 22 [3], [8], [9], [12]. The Fgf binding site is probably located in the groove that is found between the second and third Ig domain. The heparin-binding site has been localized to the second Ig domain that contains a stretch of basic amino acid residues. We have recently noticed that the extracellular domain of human FGFRL1 promotes adhesion of various cell types when coated on plastic surfaces [13]. This activity appears to be accomplished by the second Ig domain that interacts with heparan sulfate chains of glypican molecules found on most cell surfaces [13], [14].

In contrast to the extracellular domain, the intracellular part of FgfrL1 is unique and does not contain any tyrosine kinase domain as typically found in the classical Fgfrs. It is much shorter, in the case of mouse FgfrL1 it is only 134 amino acid residues in length [3], [15], and does not share much similarity with any other protein [4]. Since FgfrL1 lacks the tyrosine kinase activity, several researchers have speculated that it might function as a decoy receptor that would interact with Fgf ligands and sequester them away from the classical Fgfrs [3], [9], [12]. Although attractive, this hypothesis was recently challenged by experiments with gene arrays, which suggested that FgfrL1 might exert a positive, rather than a negative effect on Fgf signaling. FgfrL1 knock-out mice did not show any compensatory effect in the expression levels of typical Fgf signaling targets as would be expected when a negative regulator is deleted [16].

We therefore tried to elucidate the mechanism, by which FgfrL1 might participate in signaling. When we aligned the intracellular sequences of FgfrL1 from different vertebrates in a multiple sequence alignment, we identified several conserved motifs and elements, although the aligned sequences show very low overall sequence identity [4], [8], [17]. First, there are a few basic residues in the juxtamembrane region that are probably required for insertion of the polypeptide in correct orientation into the cell membrane [18]. Then, there is a dileucine motif that in other proteins has been shown to act as a mediator of endocytosis and transmembrane trafficking [19], although experimental evidence for a similar function in FgfrL1 is missing. Following the dileucine sequence, there are two tyrosine-based motifs YXXΦ arranged in tandem (PKLYPKLYTDV). Similar tyrosine-based motifs are found in regulatory proteins with high turnover rate where they mediate internalization and segregation to endosomes and lysosomes [19]. Finally, there is a histidine-rich sequence at the C-terminus of FgfrL1 where 5-10 histidine residues alternate with threonine, serine and cysteine residues. This sequence can interact with zinc ions, as recently demonstrated by atomic absorption [17].

In a previous publication, we carefully analyzed the function of the tandem YXXΦ motif and the histidine-rich sequence [8]. We found that both motifs control the turnover rate of FgfrL1. When they were mutated or deleted, either alone or in concert, the modified proteins stayed for a prolonged period of time at the cell membrane where they might interact with Fgf ligands. In sharp contrast, the wild-type protein was barely found at the plasma membrane but rather in the trans-Golgi compartments and in intracellular vesicles. Constructs with a truncated C-terminus (FgfrL1ΔC) lacking the dileucine peptide, the YXXΦ motifs and the histidine-rich sequence therefore proved to be valuable tools to study the distribution of FgfrL1 and its interaction with Fgf ligands in cell culture experiments [9]. Interestingly, we also identified a human patient with a craniosynostosis syndrome who suffered from a frameshift mutation in the region corresponding to the C-terminal end of FGFRL1 [8]. This frameshift mutation removed part of the histidine-rich sequence and compromised the turnover rate of the protein as verified in cell culture experiments.

It is important to emphasize that FgfrL1 expression has been demonstrated only at the mRNA level, utilizing sensitive techniques such as Northern blotting [1]–[3], qPCR [6]–[7] and in situ hybridization [6]–[7]. So far, we have not been able to show expression of endogenous FgfrL1 protein under physiological conditions, be it with a palette of eight different monoclonal antibodies or with several polyclonal antisera [8]. This fact illustrates that the levels of endogenous FgfrL1 protein must be very low and/or that the protein has a very rapid turnover rate. Nevertheless, expression of FgfrL1 protein could be demonstrated in cell culture experiments by indirect immunofluorescence [8] and Western blotting [9] after over-expression of FgfrL1 cDNA clones containing a strong CMV promoter.

In the present study, we analyzed the function of the intracellular motifs in a mouse model. To this end, we constructed a knock-in mouse, in which the C-terminal end of FgfrL1, including the dileucine peptide, the tandem YXXΦ motif as well as the histidine-rich sequence, was replaced by GFP. We expected that this modification should cause a delay in the turnover of the protein and consequently an over-activity of FgfrL1 (gain of function) as compared to our conventional knock-out mice, which show loss of FgfrL1 function.

## Materials and Methods

### Cloning of FgfrL1ΔC-GFP cDNA

A modified mouse FgfrL1 cDNA was prepared. For this purpose, a 1334 bp cDNA fragment covering nucleotides 81–1414 of the mouse FgfrL1 mRNA (AJ293947) [15] corresponding to amino acids 1-440 was inserted in-frame into the EcoRI-BamHI site of the GFP expression vector pEGFP-N3 (Clontech). The encoded FgfrL1ΔC-GFP fusion protein comprised the extracellular domain, the transmembrane domain and 46 amino acids of the intracellular domain, but lacked the dileucine sequence, the two lysosomal targeting motifs YXXΦ as well as the histidine-rich sequence. Authenticity of the construct was confirmed by DNA sequencing.

### Generation and characterization of mutant mice

The genetical modification made to the cDNA sequence was also inserted into exon 7 of the mouse FgfrL1 gene (NM_054071). To this end, a targeting vector of 13,8 kb was constructed by subcloning five fragments into the pSP72 cloning vector (inGenious Targeting Laboratory, Stony Brook NY, USA). The five fragments were (i) a long homology arm (6125 bp, corresponding to positions 108,699,954–108,706,078 of NC_000071.6), (ii) the in-frame GFP sequence (729 bp), (iii) a middle arm (886 bp corresponding to positions 108,706,346–108,707,231), (iv) the pGK-gb2 LoxP/FRT-flanked neo-cassette (1711 bp) and (v) a short homology arm (1949 bp corresponding to positions 108,707,232–108,709,180). The homology arms were derived from a C57BL/6 BAC clone that comprised the FgfrL1 gene (RP23: 56M23). Authenticity and reading frame of the final targeting vector were confirmed by restriction analysis and DNA sequencing.

The targeting vector was linearized with NotI and electroporated into hybrid embryonic stem cells (C57BL/6x129/SvEv). After selection with G418, resistant clones were expanded and analysed by PCR for homologous recombination. Five positive clones were further confirmed by Southern blotting with probes specific to the long and the short arm of the targeting vector, respectively. The sequence of the inserted cassette encompassing exon 7, GFP and 3'UTR was also verified by DNA sequencing of a PCR fragment amplified from the genomic DNA of the clones.

Positive embryonic stem cells were microinjected into C57BL/6 blastocysts and implanted into pseudo-pregnant foster mice. Resulting chimeras with agouti coat color were mated to C57BL/6-FLP mice in order to remove the neo-cassette. Genomic DNA from tail or ear biopsies of offspring was genotyped by PCR. One male and one female that had the neo-cassette deleted were further bred until the 8th generation by mating with C57BL/6 mice. Preparation of conventional FgfrL1 knock-out mice has been described elsewhere [5].

This study was carried out in strict accordance with the recommendations of the Swiss federal authorities. Generation and breeding of the animals were approved by the "Amt für Landwirtschaft und Natur", Kanton Bern, Switzerland (Permit number BE84/12). All efforts were made to minimize suffering. Littermates of wild-type and genetically modified animals were used for comparative studies. Mice were euthanized with CO_2_ according to the national guidelines. Homozygous FgfrL1ΔC-GFP knock-in mice did not suffer because they are phenotypically normal (see Results). Homozygous FgfrL1 knock-out animals, which are perinatally lethal, were analyzed exclusively between embryonic stages E14.5 and E18.5 after euthanasia. No *in vivo* manipulations, such as surgery or injection, were carried out.

### Cell culture and transfection

Human embryonic kidney cells HEK293 (ATCC CRL-1573) were maintained at 37°C under a humidified atmosphere containing 5% CO_2_ in Dulbecco's Modified Eagle Medium (DMEM) supplemented with 10% fetal bovine serum, 100 units/ml penicillin, 100 µg/ml streptomycin and non-essential amino acids. Cells were grown on cover slips in 12-well plastic dishes. Subconfluent cultures were transfected with DNA constructs using Metafectene according to the manufacturer's instructions (Biontex, Martinsried, Germany).

### RNA isolation and Northern blotting

Tissues were dissected from the embryos, incubated in RNAlater (Sigma) and stored until use. Samples were re-suspended in RNA lysis buffer (Qiagen) and homogenized with a Polytron (Kinematica, Switzerland). RNA was prepared using the GeneElute mammalian total RNA kit (Sigma) and separated under standard conditions [20] on agarose gels in the presence of 1 M formaldehyde. Fragments resolved on the gel were transferred to a nylon membrane by vacuum blotting. The membrane was hybridized overnight at 42°C with radioactively labeled cDNA probes in a buffer containing 50% formamide [20]. Following hybridization, the blot was washed with standard saline citrate (SSC) and exposed to X-Ray film (Sigma).

Hybridization probes were prepared by digestion of plasmids containing FgfrL1 (pcDNA3.1) [15] or GFP (pEGFP-N3, Clontech). The final probes encompassed nucleotides 81–1705 (1625 bp) of FgfrL1 (accession number AJ293947) and nucleotides 642–1399 of GFP (accession number U57609), respectively. These fragments were labeled by the random primed oligolabeling method with [á-^32^P] dCTP [21].

### Real-time PCR

Total RNA was transcribed into first-strand cDNA using random hexamer primers and Improm-II Reverse Transcriptase (Promega). Aliquots were analyzed by PCR with the 7500 Fast Real-Time PCR System (Applied Biosystems). The final reaction mixtures contained 5 µl of SYBRgreen (Life Technologies) and 500 nM of each primer (Table S1) in a total volume of 10 µl.

### Whole-mount in situ hybridization

In situ hybridization was performed as previously described [22] using E15.5 mouse kidneys. A riboprobe specific for FgfrL1 was prepared by transcription of the mouse cDNA sequence in the presence of digoxigenin-labled UTP using the SP6/T7 DIG RNA Labeling kit from Roche.

### Histology

Tissues were excised and fixed with 4% paraformaldehyde (PFA) in PBS at 4°C. After one day, the samples were dehydrated by passing them through a graded series of ethanol, isoproanol and xylol. The specimens were embedded in paraffin (Shandon Citadele 1000 Tissue Processor, Thermo Fisher Scientific) and cut to 4 µm sections. The sections were rehydrated by incubation in xylol and carrying them through a graded series of ethanol (100%, 90%, 80%, 70%, 50%). After staining with hematoxylin (Sigma) for 3 minutes, the slides were cleared in 70% ethanol, 0.5% acetic acid and rinsed with water. Counter-staining was performed with eosin (Sigma) for 2 minutes. Finally, the slides were dehydrated with ethanol, incubated in xylol and mounted in Entellan (Merck).

### GFP epifluorescence

Selected tissues were dissected out of the embryos at different developmental stages and fixed overnight in 4% PFA, or alternatively, for 20 min in methanol/acetone (1∶1 v/v). For paraffin sections, the samples were dehydrated in ethanol and embedded in paraffin. For cryosections, the samples were embedded directly in Tissue Tek O.C.T. compound (Sakura, The Netherlands). Sections of 10 µm were cut and inspected under a microscope equipped with epifluorescence optics (Nikon Eclipse E800).

### Immunohistochemistry

Paraffin sections of 4 µm were re-hydrated and boiled for 5 min in 10 mM citric acid, 0.05% Tween 20, pH 6.0 to unmask antigenic determinants. After three washing steps in TBST (Tris buffered saline (TBS) containing 0.025% Tween 20), cell membranes were permeabilized with 0.2% Triton X-100 for 10 min. Unspecific sites were blocked with 5% bovine serum albumin in TBS. Incubation with the primary antibody was performed in TBST overnight. After washing, the sections were incubated with the secondary antibody for 1 h. Nuclei were stained for 3 min with 4',6-diamidin-2-phenylindol (DAPI) in TBST. Finally, the slides were mounted with Aquatex (Merck).

The following primary antibodies were used: rabbit polyclonal anti-insulin (Santa-Cruz, sc-9168, diluted 1∶200), rabbit monoclonal anti-insulin (Cell Signaling Technology, C27C9, 1∶200), monoclonal humanized anti-FGFRL1 (Fab, final concentration 1 µg/ml) [8], polyclonal goat anti-FGFR5 (Santa Cruz Biotechnology, Inc., 1∶200 and R&D Systems, 1∶2000), polyclonal rabbit anti-GFP antibody (Clontech, 1∶1000), polyclonal rabbit anti-GFP (MBL, Woburn, MA, USA 1∶200), GFP-Booster (Chromotek, Martinsried, Germany 1∶200), mouse monoclonal anti-GFP (Roche, 1∶500), monoclonal mouse anti-GAPDH (Thermo Fisher Scientific, 1∶2000).

To detect bound primary antibodies, the following secondary antibodies were employed: goat anti-rabbit IgG alkaline phosphatase conjugate, bovine anti-goat IgG alkaline phosphatase conjugate, goat anti-mouse IgG alkaline phosphatase conjugate (all from Sigma, 1∶10,000), goat anti-human IgG (Fab) alkaline phosphatase conjugate (Jackson Laboratories Inc., 1∶20,000), Cy3-conjugated goat anti-mouse IgG and Cy3-conjugated goat anti-human IgG (Fab) (Jackson Laboratories Inc., 1∶400), anti-rabbit IgG coupled to TRITC (Sigma, 1∶1000), IRDye 680 donkey anti-mouse IgG (Li-Cor, Lincoln, NE, USA, 1∶15,000).

### Skeletal preparations

Whole mount skeletons were stained according to the method of McLeod [23]. Skeletal elements were dissected and fixed in ethanol. After one week, the ethanol was replaced by acetone in order to remove remaining fat tissue. Three days later, the samples were stained with a solution containing 0.03% alcian blue 8GS, 0.02% alizarin red S, 7% v/v acetic acid and 50% v/v ethanol. One week later, the specimens were cleared with 1% KOH and stored in glycerol.

### Gel electrophoresis and Western blots

Kidneys were dissected from embryos and directly dissolved in hot SDS sample buffer containing proteinase inhibitors (2 mM PMSF, 5 mM EDTA). Proteins were separated under standard conditions on 10% SDS polyacrylamide gels and transferred onto nitrocellulose membranes by semi-dry blotting (Trans-Blot SD, Biorad). Unspecific sites on the membranes were blocked with 5% milk powder in PBS. The membranes were incubated with the primary antibody overnight at 4°C and then with alkaline phosphatase-conjugated secondary antibodies for 1 h at room temperature. Bound antibodies were detected by reaction with 5-bromo-4-chloro-3-indolyl-phosphate and nitro blue tetrazolium substrate. Alternatively, the blots were incubated with an IRDye 680 labeled secondary antibody and analyzed with the Li-Cor Odyssey Infrared Imaging system (Li-Cor, Lincoln NE, USA).

## Results

### FgfrL1ΔC-GFP remains at the cell membrane

**Figure 1:**
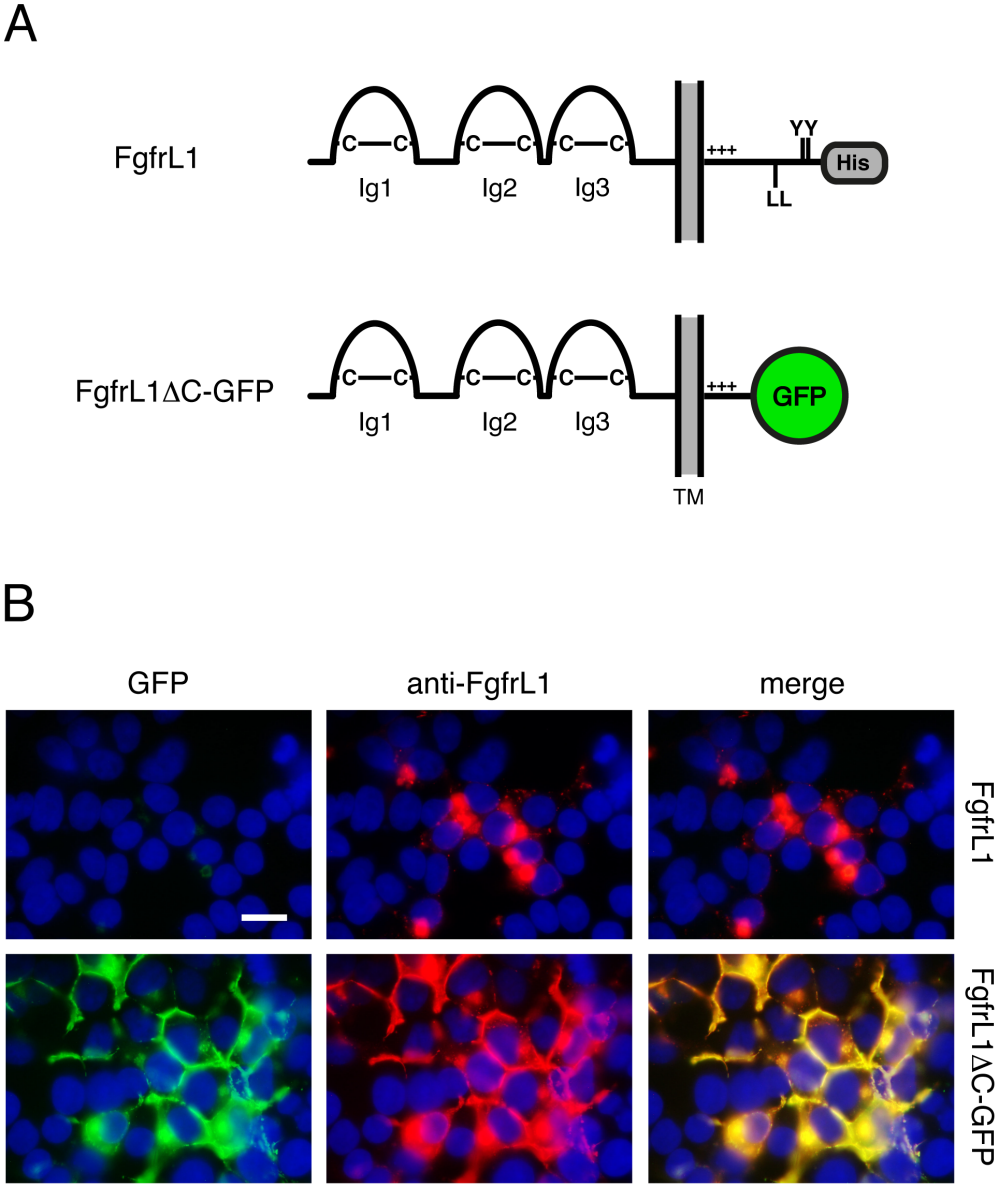
FgfrL1ΔC-GFP protein remains at the plasma membrane. A) Schematic drawing of full-length FgfrL1 and of truncated FgfrL1ΔC-GFP. The three Ig domains with disulfide bridges (C–C), transmembrane domain (TM), positively charged juxtamembrane region (+++), dileucine motif (LL), tandem tyrosine-based motif (YY), histidine-rich region (His) and GFP moiety are indicated. B) HEK293 cells were transfected with constructs coding for full-length FgfrL1 and for FgfrL1ΔC-GFP as indicated. After one day, the cells were fixed and stained with a monoclonal antibody against FgfrL1. Full-length FgfrL1 was localized primarily to intracellular structures, whereas the mutant protein was found mainly at the cell membrane. The signal from GFP (green) and from the antibody (red) colocalized as demonstrated by superimposition of the two panels. Cell nuclei were stained with DAPI (blue). Bar = 20 µm.

Our aim was to generate genetically modified mice lacking all the conserved motifs of the intracellular FgfrL1 domain. Since this was a complex endeavor, we first confirmed that the designed construct was properly expressed in cultured cells. For this purpose, we prepared an expression vector with the mouse FgfrL1 cDNA sequence lacking the region corresponding to amino acids 441–529 and instead containing the in-frame sequence for GFP. This construct coded for an FgfrL1ΔC-GFP fusion protein lacking the intracellular dileucine peptide, the two YXXΦ motifs and the histidine-rich sequence (Fig. 1A). One day after transfection of the construct into HEK293 cells, we observed strong epifluorescence from GFP at the cell membrane, especially at contact sites where two cells touched each other, in addition to some fluorescent signal at intracellular structures (Fig. 1B). When stained with a monoclonal antibody against FgfrL1, the GFP signal overlapped to a large extent with the signal of the antibody. In contrast, full-length FgfrL1, which was included in our experiment as a control, was barely found at the plasma membrane, but localized primarily to intracellular structures, as demonstrated with our monoclonal antibody. Thus, the mouse FgfrL1ΔC-GFP construct showed the same subcellular distribution as the human FGFRL1ΔC construct described in a previous publication [8]. This result suggested that the conserved motifs of the intracellular domain also control the turnover rate of mouse FgfrL1.

### Generation of FgfrL1ΔC-GFP mice

**Figure 2:**
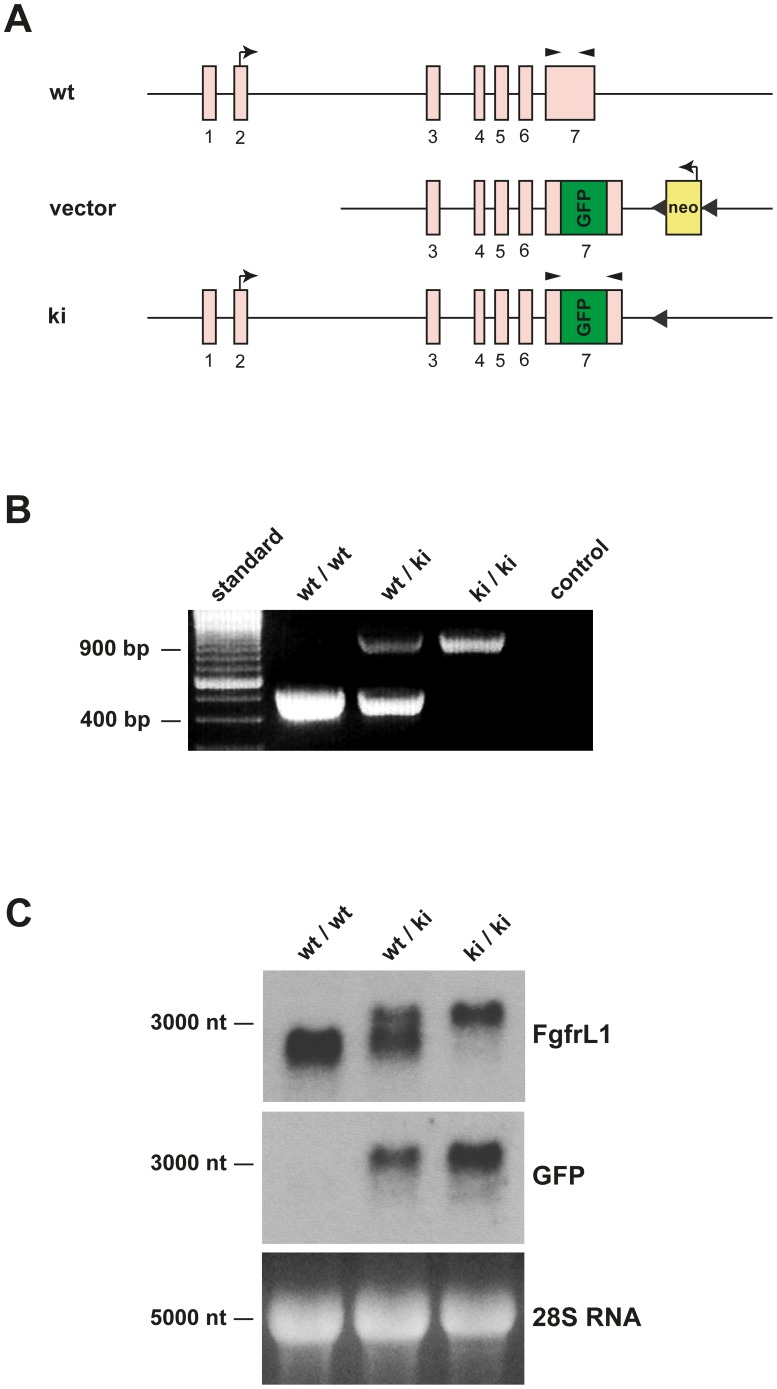
Generation and identification of knock-in mice. A) Construct and targeted genomic DNA before and after homologous recombination. The region of exon 7, which codes for the dipeptide sequence, the two YXXΦ motifs and the histidine-rich sequence (amino acid residues 441–529), was replaced by an in-frame GFP sequence. An FPL-flanked neo cassette was inserted downstream of the FgfrL1 gene to enable selection of positive clones by G418. This cassette was subsequently removed by mating with FLP-mice. Kinked arrows show the translation initiation sites of FgfrL1 and Neo, respectively. Arrowheads show the relative position of primers used for genotyping. The sketch is not drawn to scale. B) Genotyping of mutant mice by PCR. Samples from wild-type animals yielded a unique fragment of 504 bp, whereas samples from knock-in mice gave a unique fragment of 966 bp. A sample lacking any genomic DNA (negative control) and a DNA standard were included on the gel. C) Northern blots with samples from mutant mice. Total RNA from the tongue of wild-type, heterozygous and homozygous knock-in mice at E18.5 was separated on an agarose gel, transferred to a Nylon membrane and hybridized with radiolabeled probes for FgfrL1 and GFP, respectively. The 28S RNA stained with ethidium bromide was included as a loading control.

In a second step, the analogous mutation was introduced into the mouse genome. To this end, a targeting vector was constructed, in which the codons for amino acids 441–529 were deleted from exon 7 and replaced by the sequence for GFP (Fig. 2A). An FLP-flanked neo cassette was introduced 282 nucleotides downstream from the end of the FgfrL1 gene in order to allow selection of positive clones by G418. The final targeting vector was introduced into embryonic stem cells and used to generate FgfrL1ΔC-GFP knock-in mice by homologous recombination. Offspring with the correct genotype were crossed with C57BL/6-FLP mice to remove the neo cassette. Finally, homozygous knock-in mice were obtained by mating pairs of heterozygous FgfrL1ΔC-GFP mice.

Homozygous FgfrL1ΔC-GFP mice could be distinguished from heterozygous and wild-type mice by diagnostic PCR (Fig. 2B). Amplification with a pair of primers that annealed to regions of exon 7 outside of the GFP cassette produced characteristic bands of 966 bp from the recombinant allele and 504 bp from the wild-type allele. The mutant allele was correctly transcribed and spliced as demonstrated on a Northern blot using total RNA from tongue (Fig. 2C). Hybridization with a probe for mouse FgfrL1 produced a band of 2900 nucleotides with RNA from wild-type mice and a band of 3400 nucleotides with RNA from homozygous knock-in mice. Heterozygous mice yielded two bands, each with half the intensity of the bands from homozygous mice. Hybridization with a probe for GFP produced a single band of 3400 nucleotides. This band was obtained with full intensity from homozygous knock-in mice and with half the intensity from heterozygous mice, but not at all from wild-type mice. Taken together, these results confirmed that the mutated allele was correctly transcribed into FgfrL1ΔC-GFP mRNA. We also transcribed this mRNA into cDNA and confirmed, by DNA sequencing, correct splicing of exon 6 to exon 7 and in-frame ligation to GFP.

**Figure 3:**
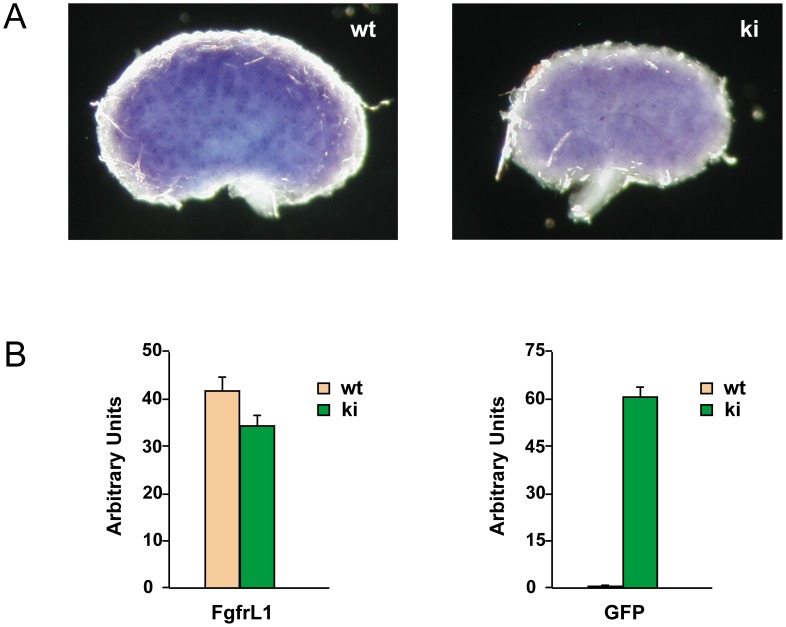
Expression of FgfrL1ΔC-GFP in the developing kidney. A) Kidneys from wild-type and FgfrL1ΔC-GFP knock-in mice at stage E15.5 were stained by whole-mount in situ hybridization with riboprobes for FgfrL1. The resulting patterns of wild-type and knock-in samples looked highly similar. B) Quantification of FgfrL1ΔC-GFP expression in kidneys of E17.5 by RT-PCR using primer pairs specific for FgfrL1 and GFP, respectively. No significant differences in FgfrL1 mRNA levels could be observed between samples from wild-type and knock-in mice. On the other hand, GFP was expressed exclusively in kidneys from knock-in mice as expected.

Expression of the FgfrL1ΔC-GFP construct was confirmed in developing kidneys. By whole-mount in situ hybridization of E15.5 kidneys (Fig. 3A) we observed a dotted pattern very similar to the pattern previously described in wild-type mice [22]. By RT-PCR, we detected FgfrL1 expression at similar levels in wild-type and knock-in kidneys of developmental stages E15.5 and E17.5 (Fig. 3B). However, expression of the GFP cassette was observed exclusively in knock-in kidneys as expected.

Heterozygous knock-in mice were bred in the C57BL/6 background until the 8th generation. To our surprise, heterozygous as well as homozygous knock-in mice did not differ phenotypically from wild-type mice. They were viable, fertile and did not display any obvious malformations. Male and female offspring were obtained in similar numbers. The mutated allele segregated according to the Mendelian law (Table 1). Crossing a pair of heterozygous FgfrL1ΔC-GFP mice yielded wild-type, heterozygous and knock-in mice with the expected 1∶2∶1 ratio. We also mated heterozygous FgfrL1ΔC-GFP mice with heterozygous FgfrL1 knock-out mice and obtained in this way mutant mice carrying a single FgfrL1ΔC-GFP allele. Even these mice were viable, fertile and phenotypically normal. As of today, one homozygous FgfrL1ΔC-GFP male has been living in the animal facility without pathological findings for more than 18 months; a knock-out/knock-in male with a single FgfrL1ΔC-GFP allele has been living there for more than 21 months. These results suggested that the conserved intracellular motifs of mouse FgfrL1 are not required for survival and that one truncated allele is sufficient for normal life.

**Table 1:** Transmission of the knock-in mutation.

	het x het (n = 214)	hom x wt (n = 68)
**wt/wt**	**26.6%**	**0%**
**wt/ki**	**50.0%**	**100%**
**ki/ki**	**23.4%**	**0%**

### Detection of FgfrL1ΔC-GFP fusion protein

**Figure 4:**
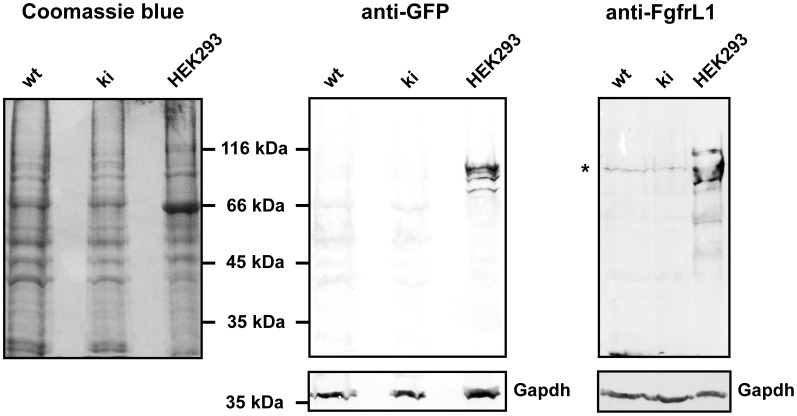
Western blot with FgfrL1ΔC-GFP fusion protein. Kidneys were dissected from wild-type and knock-in mice of stage E15.5 and immediately dissolved in hot SDS sample buffer containing proteinase inhibitors. The protein extracts were separated on 10% polyacrylamide gels and transferred onto nitrocellulose membranes. The blots were incubated with a monoclonal antibody against GFP, a monoclonal antibody against Gapdh and polyclonal antibodies against FgfrL1 as indicated. Bound antibodies were visualized with secondary alkaline phosphatase-conjugated antibodies. As a control, an extract from HEK293 cells transfected with the FgfrL1ΔC-GFP construct was included. The GFP signal could be detected only in the control lane with protein extract from transfected cells, but not in the lanes containing extracts from kidneys of wild-type and knock-in mice. The GFP-positive bands also reacted with polyclonal anti-FgfrL1 antibodies. Note that the FgfrL1ΔC-GFP fusion protein migrates as several bands with a molecular mass of 75–85 kDa due to glycosylation [13]. The band marked by an asterisk in the panel stained with anti-FgfrL1 antibodies most likely represents a cross-reacting protein because it migrates with the same mobility in the lanes containing wild-type and knock-in protein extracts, although wild-type FgfrL1 has a molecular mass of 67 kDa, whereas GFP knock-in protein has a molecular mass of 85 kDa (after glycosylation).

Since the three conserved motifs of the intracellular FgfrL1 domain had been replaced by a GFP cassette, we tried to detect expression of the fusion protein by epifluorescence emitted from the GFP moiety. However, when we inspected cryosections prepared from kidney or tongue between developmental stages E15.5 and P30, we could not detect any signal under the fluorescence microscope. We therefore attempted to amplify the signal with antibodies directed against GFP. But again, we did not observe any signal, although we tested antibodies from four different suppliers. Finally, we turned to Western blotting and tried to identify the GFP fusion protein after separation on SDS polyacrylamide gels (Fig. 4). In this way, we were able to detect FgfrL1ΔC-GFP that had been over-expressed in HEK293 cells and included in the experiment as a positive control, but we never detected any signal with samples obtained from kidney or tongue of our knock-in mice at various developmental stages. Loading more protein onto the gel or amplification of the signal with a fluorescent secondary antibody followed by detection with a Li-Core imaging system did not help. Thus, we had to conclude that the levels of the endogenous FgfrL1ΔC-GFP protein were too low to be detected by biochemical means, although expression could be confirmed at the level of the mRNA by Northern blotting and RT-PCR (Figs. 2 and 3).

### Mutant kidneys show a slight reduction in the number of glomeruli

**Figure 5:**
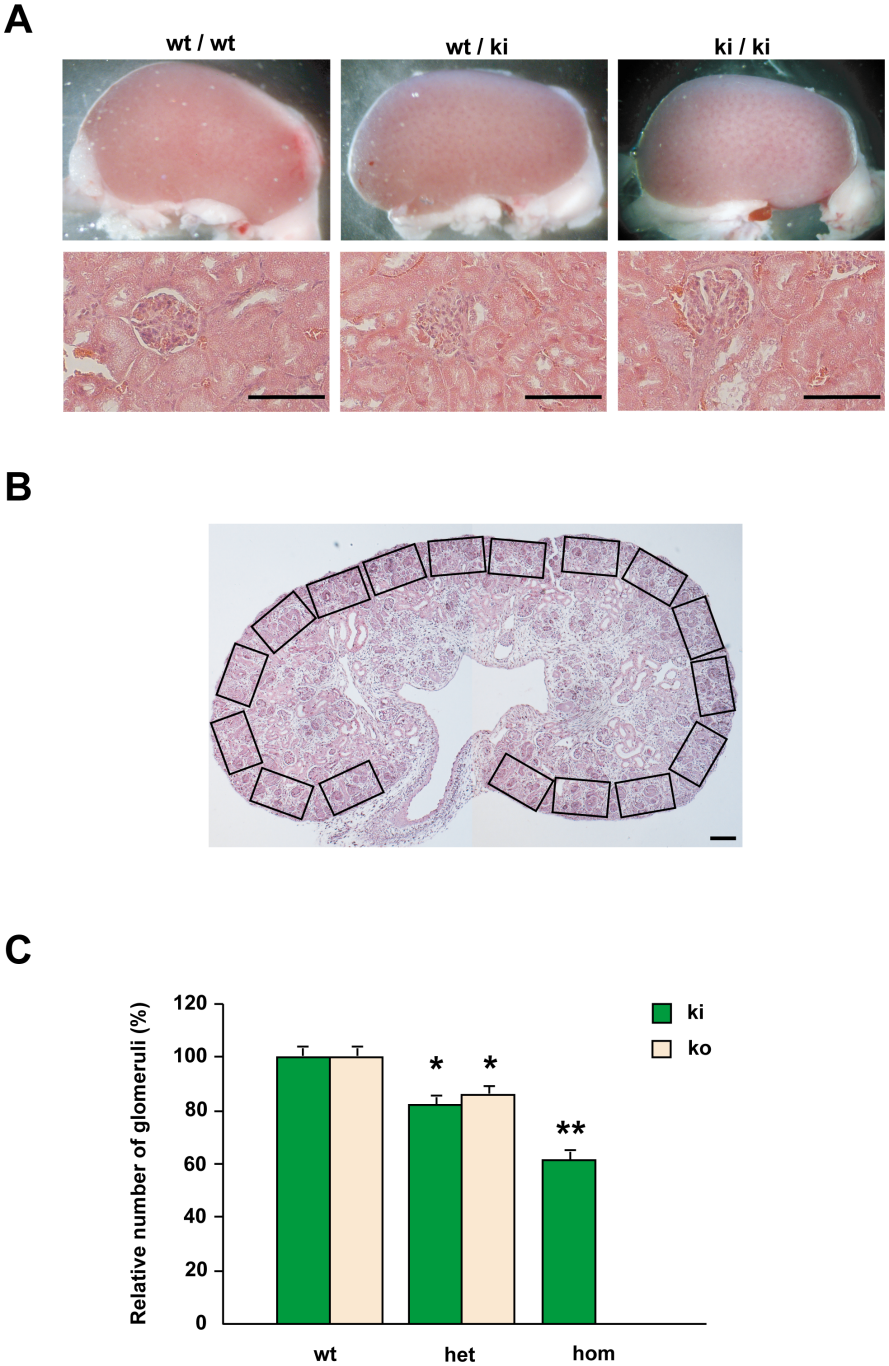
Morphology of kidneys. A) Kidneys from wild-type and mutant mice did not reveal any alterations in overall appearance two month after birth. Likewise, no difference was observed on paraffin sections stained with H&E. Bar = 100 µm. B) Example of a thin section stained with H&E to demonstrate the strategy used to determine the number of glomeruli in kidney samples of E17.5. The number of glomeruli within each rectangle was counted under higher power magnification. Subsequently, the number of glomeruli per mm^2^ was calculated for each sample. C) Statistical analysis of the number of glomeruli in wild-type, heterozygous and homozygous kidneys at E17.5. Numbers are given in relation to the numbers of wild-type kidneys that were arbitrarily set to 100%. Homozygous FgfrL1ΔC-GFP, heterozygous FgfrL1ΔC-GFP and heterozygous FgfrL1 knock-out mice showed a slight, but significant reduction in the total number of glomeruli (* p<0.05 Student's t-test). The numbers of individual kidneys, which were analyzed in this fashion, were: wild-type (for knock-in littermates) 7, wild-type (for knock-out littermates) 5, heterozygous knock-in 17, heterozygous knock-out 7, homozygous knock-in 8. Homozygous knock-out animals do not develop any metanephric kidneys. Note that number of kidneys equals number of animals since only one kidney per animal was used for analysis.

Since FgfrL1 knock-out mice lack the metanephric kidneys [7], we specifically focused on the kidneys of FgfrL1ΔC-GFP mice. Histological examination after H&E staining showed that the metanephric kidneys developed normally. Size and morphology of kidneys from wild-type, heterozygous and homozygous knock-in mice were comparable (Fig. 5A). Only when we counted the exact number of glomeruli from E17.5 embryos (Fig. 5B), we observed a slight, but significant reduction in the mutant kidneys. Some reduction could also be noted in the kidneys from heterozygous animals. Interestingly, a similar reduction was even found in kidneys from heterozygous FgfrL1 knock-out mice (Fig. 5C). These results suggested that manipulating the copy number of FgfrL1 or the length of the intracellular domain slightly affect the development of the metanephric kidneys. However, the differences are just minor and influence neither survival nor overall phenotype of the animals.

### The diaphragm is normal

**Figure 6:**
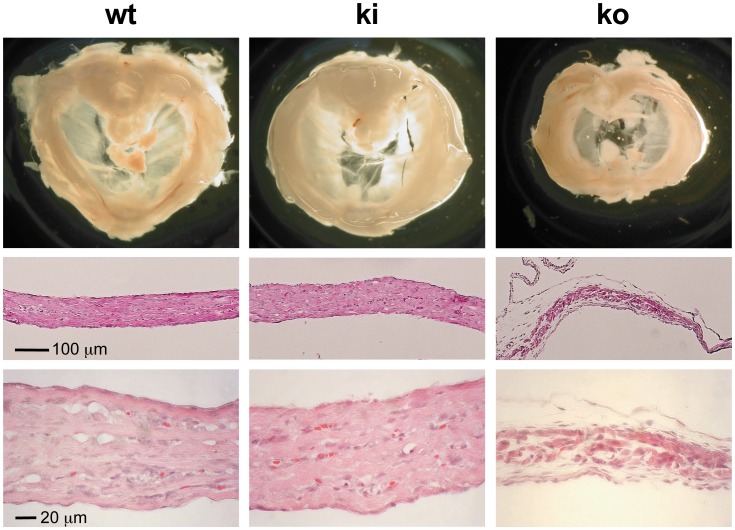
Morphology of diaphragms from mutant mice. Diaphragms from wild-type, homozygous FgfrL1ΔC-GFP knock-in and homozygous FgfrL1 knock-out mice were compared at E18.5. Coronal sections of the costal muscles stained with H&E are depicted below the panels showing overall morphology. Diaphragms from wild-type and knock-in mice did not reveal any differences. However, the diaphragms from knock-out animals were smaller and significantly thinner. Bar = 100 µm.

FgfrL1 knock-out mice die at birth due to a malformed diaphragm muscle that is too weak to inflate the lungs after delivery [5]. When we inspected the diaphragm of our FgfrL1ΔC-GFP mice we did not find any histological abnormalities at E18.5 (Fig. 6). In fact, when stained by H&E, the costal muscles displayed nice bundles of muscle fibers with a total thickness comparable to that of wild-type mice. Only the diaphragm from our conventional FgfrL1 knock-out mice was consistently smaller and significantly thinner than that from wild-type mice as previously reported (Fig. 6) [5].

### No skeletal malformation

**Figure 7:**
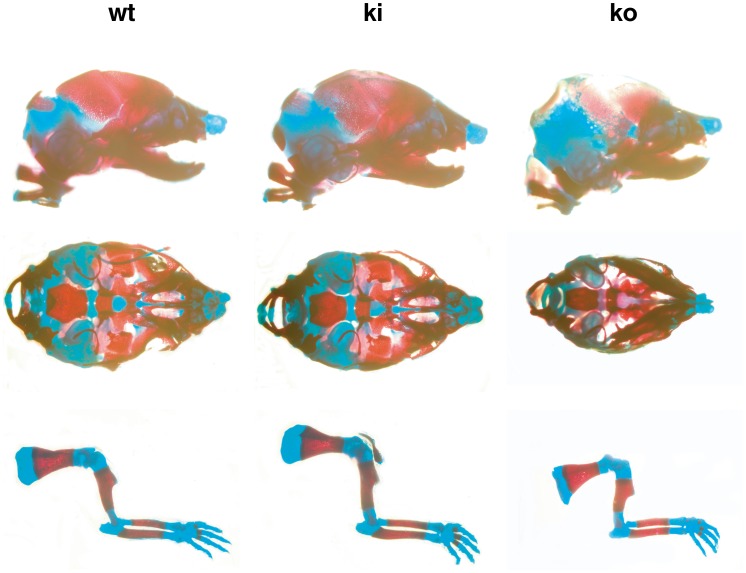
Morphology of skeletal elements. Skull and long bones from wild-type, homozygous knock-in and homozygous knock-out mice at E18.5 were stained with alcian blue (specific for cartilage) and alizarin red (specific for mineralized tissues). Skeletal elements from FgfrL1ΔC-GFP knock-in mice did not show any abnormalities when compared to wild-type mice. Only samples from FgfrL1 knock-out animals displayed some alterations. The long bones were frequently shorter and the skulls were often smaller than those from wild-type animals. Bar = 100 µm.

In a previous publication, we observed some skeletal malformations with our FgfrL1 knock-out mice, such as a dome-shaped skull [8]. Moreover, another group described hypoplasia of most skeletal elements in FgfrL1 knock-out mice, including a shortened skeleton, malformed vertebrae, thinner calvaria and delayed fusion of bones at the cranial base [6]. We therefore analyzed skeletal preparations of our FgfrL1ΔC-GFP mice after staining with alcian blue and alizarin red (Fig. 7). However, compared to wild-type littermates of E18.5, we could not detect any significant differences in size and shape of bones (red) and cartilage (blue) of skulls and limbs. This result suggested that the processes of membranous and endochondral ossification were not affected by the lack of the conserved intracellular motifs of FgfrL1. However, FgfrL1 knock-out mice, which were included in our analysis for comparison, did show a slight reduction in the overall size of the skeleton (Fig. 7). The skull was somewhat smaller and the calvaria were hypomineralized, consistent with previous reports [6], [8].

### Production of insulin by pancreatic β-cells

**Figure 8:**
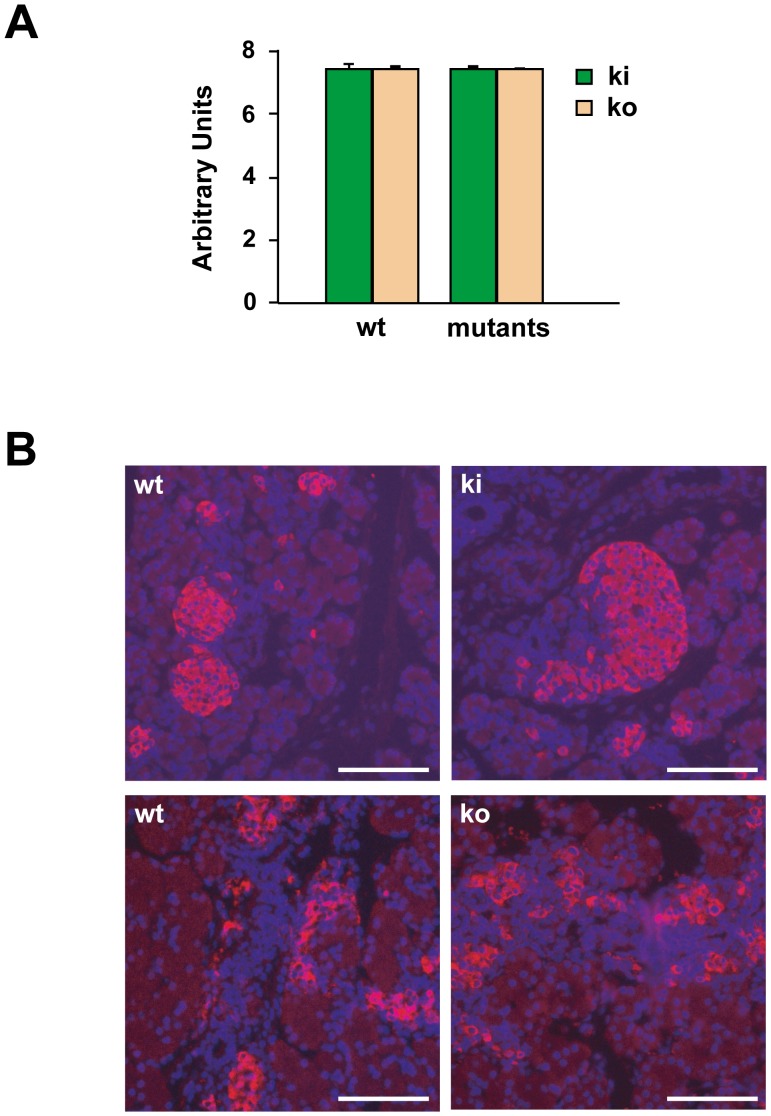
Production of insulin by pancreatic β-cells. A) Quantitative RT-PCR of insulin mRNA in the pancreas from wild-type and mutant mice. Total RNA was isolated at E18.5 and transcribed into cDNA. When normalized to the expression of RpS9, there was no difference in insulin expression between samples from wild-type, homozygous knock-in and homozygous knock-out samples. The bars give the mean of triplicate measurements with standard deviation. B) Localization of insulin in islets of the pancreas from E18.5 mice. Paraffin sections of the pancreas from wild-type, homozygous knock-in and homozygous knock-out mice were incubated with a monoclonal antibody against murine insulin, followed by a rhodamine-labeled secondary antibody. No qualitative difference was noted between samples from wild-type and mutant mice. Bars = 100 µm.

It has been published that human FGFRL1 is expressed at relatively high levels in the pancreas [2]. With polyclonal antibodies, mouse FgfrL1 was subsequently localized to insulin secretory granules of pancreatic β-cells [24]. These researchers also reported that human FGFRL1 would enhance the production of insulin by murine βTC3 cells as determined by ELISA. We therefore analyzed the expression of insulin in our knock-out and knock-in mouse models (Fig. 8). By quantitative RT-PCR, however, we did not observe any alterations of insulin mRNA levels in the pancreas of E18.5 mice, neither in the homozygous FgfrL1ΔC-GFP mice nor in the FgfrL1 knock-out mice. Consistent with this observation, we detected comparable levels of insulin with a monoclonal antibody on thin sections of pancreas from wild-type, knock-in and knock-out mice. Moreover, our genetically modified mice never showed any signs of hyperglycemia as determined by hexokinase/glucose-6-phosphate dehydrogenase (Roche). Thus, the murine FgfrL1 does not appear to affect the production of insulin by pancreatic β-cells.

## Discussion

FgfrL1 is the most recently discovered member of the Fgfr family [4]. It is involved in the formation of kidneys and diaphragm as demonstrated with genetically modified mice. Homozygous FgfrL1 knock-out mice die at birth [5], [6]. They lack both kidneys [7] and show an underdeveloped diaphragm that is too weak to inflate the lungs after birth [5]. In spite of these vital functions, the molecular mechanisms, by which FgfrL1 controls organogenesis, are not known.

Interestingly, the sequence of the extracellular domain of FgfrL1 is well conserved among different species, while that of the intracellular domain is not [4]. There are only a few conserved motifs in the intracellular domain that are found in most animals [8]. These include (i) a dileucine sequence that occurs 7–9 residues downstream of two acidic residues, (ii) two tyrosine-based motifs that are arranged in tandem, and (iii) a histidine-rich sequence that contains several His-Ser and His-Thr repeats. In a previous publication we have demonstrated that the histidine-rich sequence and the tyrosine-based motifs accelerate protein turnover by targeting FgfrL1 to endosomes and lysosomes [8]. When either one of the two motifs was deleted or mutated, endocytosis of FgfrL1 was delayed and the proportion of the protein at the cell membrane increased. It has also been suggested that the tandem tyrosine-based motif might function as a binding site for phosphatases [24]. In this way, FgfrL1 might recruit phosphatases to the cell membrane and interfere with signaling [4].

In this publication, we analyzed the function of the intracellular domain with the help of genetically modified mice. We generated knock-in mice, in which the three conserved motifs of the intracellular FgfrL1 domain were deleted and replaced by a GFP cassette. To our surprise, these mice were viable, fertile and phenotypically normal. In particular, diaphragm, skull, limbs and pancreas did not show any significant alterations. Only the kidneys revealed a slight reduction in the number of glomeruli but this reduction did not affect life expectancy and well-being of the animals. We therefore have to conclude that the dileucine sequence, the tyrosine-based motifs and the histidine-rich sequence do not have any crucial signaling function. On the other hand, we have clearly documented the role of the tyrosine-based motif in protein turnover [8] and others have confirmed our findings [24]. We must therefore assume that mutant mice do not show any obvious phenotype when the turnover of a single protein like FgfrL1 is retarded, especially when this protein is expressed at very low levels.

There is one point that needs to be emphasized in this context. We did not delete the entire intracellular domain of FgfrL1 in our FgfrL1ΔC-GFP mice, but only the three well-conserved motifs (amino acid residues 441–529). Theoretically, a signaling molecule could still bind to the remaining part of the intracellular domain (residues 396–440) and fulfill its function. This possibility is rather unlikely because the remaining part of the intracellular domain is not conserved among different species and because it does not contain any known signaling motif [4], [8]. Naturally, we could not delete the juxtamembrane region as it controls insertion into the plasma membrane. This region contains several positively charged residues that are required during translation to insert the protein in correct orientation into the membrane [18].

Another unexpected observation of our study was the fact that we could not detect any epifluorescence from GFP, which had been inserted in-frame at the C-terminus of the FgfrL1ΔC protein. We also used anti-GFP antibodies to detect the fusion protein on Western blots or on thin sections, but without any success. This failure cannot be explained by the quality of the antibodies because we used antibodies from different suppliers that did react with FgfrL1-GFP fusion proteins when over-expressed in cell culture. We can also exclude a splicing or frame-shift error of the transcribed FgfrL1ΔC-GFP mRNA because we detected mRNA of the correct size on Northern blots. We even transcribed the mRNA, which was expressed by our mutant mice, into cDNA and verified its sequence. Thus, the endogenous FgfrL1ΔC-GFP protein must be expressed at extremely low levels that cannot be detected by biochemical means. This conclusion is in agreement with previous observations that the wild-type FgfrL1 protein is also expressed at extremely low levels in mice. The physiological levels are clearly too low to be detected with our monoclonal antibodies [8]. So far, we identified FgfrL1 protein only if the corresponding cDNA was over-expressed in cell culture from a strong CMV promoter [8]. This fact raises doubts about several recent reports, which detected FgfrL1 protein with polyclonal antibodies in mouse kidney [7], rat diaphragm [25], epithelium of human bladder [26] and stroma of esophageal tumors [27]. It is therefore conceivable that commercial polyclonal antibodies cross-react with unrelated proteins. A cross-reacting protein has in fact been detected by our Western blotting experiments presented in Fig. 4. The possibility that the truncated FgfrL1ΔC-GFP protein is not expressed at all can be excluded. If FgfrL1ΔC-GFP would not be expressed at all, the mice would die as FgfrL1 knock-out animals are 100% lethal [4]–[6]. However, our FgfrL1ΔC-GFP mice are 100% viable.

Although unexpected, our observations are in full agreement with a recent proteomics approach. Kim et al. presented a draft of the human proteome using high resolution mass spectrometry [28]. They analyzed 30 human tissues and primary cells and identified proteins encoded by 17,294 genes accounting for 84% of the total protein-coding genes in humans. Most importantly in our context, they did not detect any endogenous FgfrL1 protein. FgfrL1 must therefore be expressed at very low levels and/or exclusively in limited locations at a very specific developmental stage.

In spite of our negative results with FgfrL1ΔC-GFP mice, FgfrL1 must play an important biological role because all conventional FgfrL1 knock-out mice die immediately after birth [5], [6]. What is the molecular mechanism if the intracellular domain is not involved in signaling? We now speculate that the major function of FgfrL1 is to promote cell-cell adhesion and that this function is mainly conducted by the extracellular domain. In fact, when the extracellular domain was prepared in recombinant form and used to coat plastic dishes, it promoted adhesion of various cell types [13]. Moreover, FgfrL1 is usually observed at contact sites where two cell membranes touch each other. We have localized the cell binding activity of FgfrL1 by site-directed mutagenesis to the second Ig domain, which interacts with heparin [13]. It is conceivable that cells bind with this domain to heparan sulfate chains of cell surface proteoglycans (e.g. glypicans) on neighboring cells [14] and that this interaction can link together two cell membranes in a zipper-like fashion. This zipper-like activity is probably required when cells from the metanephric mesenchyme condense and differentiate into epithelial renal vesicles. It is also needed when myoblasts align and fuse into myotubes and mature myofibers. Renal vesicles of developing kidneys and differentiating myotubes of the diaphragm are the two major structures that show expression of FgfrL1 during development and that are severely affected in our knock-out mice [5], [7].

In conclusion, our results demonstrate that the intracellular domain of murine FgfrL1 is dispensable for organogenesis. We therefore speculate that most of the vital functions of FgfrL1 are conducted by the extracellular domain.

## Supporting Information

Table S1 **Primer sequences used in this study.**
(DOCX)
