# Supplementary material for: Targeted Disruption of the Intracellular Domain of Receptor FgfrL1 in Mice
Source: PLoS One. 2014 Aug 15;9(8):e105210. doi: 10.1371/journal.pone.0105210 (PMC4134281; doi:10.1371/journal.pone.0105210)
Supplement: Table S1 — Primer sequences used in this study. (DOCX) [file pone.0105210.s001.docx]

**Table S1.** Primer sequences used in this study.

| **Sequence** | **Strand** | **Amplicon** | **Sequence ID** |
| --- | --- | --- | --- |
| GACAAGGACCTGCCCTCATTG | + | 966 / 504bp | FgfrL1 (NM_054071) |
| CTAGCCATCGCTACATGTCACTG | - | 966 / 504bp | FgfrL1 (NM_054071) |
| ACCCAGGCTTTTGTCAAACA | + | 266bp | insulin (NM_008386) |
| CCTTAGTTGCAGTAGTTCTC | - | 266bp | insulin (NM_008386) |
| AGGTCGGTGTGAACGGATTTG | + | 355bp | Gapdh (NM_001289726) |
| GCGGAGATGATGACCCTTTTG | - | 355bp | Gapdh (NM_001289726) |
| GATCCCCGGCGTCTGTTTGAAG | + | 260bp | RpS9 (NM_029767) |
| GAGTCCAGGCGAACAATGAAGGATG | - | 260bp | RpS9 (NM_029767) |
